# Integrating HIV/AIDS and Alcohol Research

**Published:** 2010

**Authors:** Kendall J. Bryant, Steve Nelson, R. Scott Braithwaite, Deidra Roach

**Keywords:** Alcohol consumption, alcohol abuse, alcohol and other drug effects and consequences, risk factors, risky sexual behavior, human immunodeficiency virus, acquired immune deficiency syndrome

## Abstract

Many people at risk for or already infected with HIV abuse alcohol, contributing to the difficulties in preventing the spread of the infection and treating infected patients. Thus, alcohol-abusing patients may delay testing for HIV, accessing appropriate medical care, and initiating antiretroviral therapy (ART), which may hasten disease progression to full-blown AIDS. Alcohol abuse also increases the risk of HIV infection by promoting risky behaviors and counteracting efforts to minimize the risk of infection, prevent transmission of the virus to others once exposure has occurred, and reduce the risk of progression and organ or tissue injury after infection. In HIV-infected people undergoing treatment, concurrent alcohol abuse often renders treatment ineffective because patients frequently fail to adhere to the strict treatment regimens necessary to achieve control of the infection. Moreover, alcohol may interact with ART medications and exacerbate adverse effects of these medications. Future research needs to better integrate behavioral and biological research to identify strategies to prevent the spread of HIV infection in alcohol-abusing populations as well as focus on translational research to effectively implement promising approaches on a large scale.

The acquired immune deficiency syndrome (AIDS) was first recognized in 1981; subsequently, researchers determined that it was caused by infection with the human immunodeficiency virus (HIV). Since then, the disease has become a pandemic, with the virus infecting almost 60 million people worldwide, killing 25 million of them (Doran 2009). Although researchers have learned much about the nature of the virus, the course of the disease, the routes of transmission, and strategies to suppress viral replication and disease progression, the epidemic continues unabatedly, particularly in less developed countries. Even in the United States, where many prevention campaigns have been implemented and awareness of the transmission routes is relatively high, between 55,000 and 60,000 people become newly infected with HIV every year (Centers for Disease Control and Prevention [CDC] 2008*a*). With improved treatment options, HIV infection in the United States and other Western countries has evolved from an acute illness with rapid progression and death to a chronic condition with, in many cases, a life expectancy of 20 to 40 years, thanks to the knowledge gained from research. Globally, however, approximately 2 million people died from the disease in 2008 (Doran 2009).

Nevertheless, many challenges remain in preventing both infection with the virus and progression of the disease. One of the many factors contributing to the difficulties of preventing the spread of the infection and treating infected patients is the acute or chronic alcohol use of people who are at risk for infection or who already are infected. The alcohol–HIV/AIDS literature has grown extensively over the past 15 years, and a variety of recent reviews of this literature have summarized the interactions of alcohol use and HIV/AIDS in behavioral, biological, and biomedical areas (e.g., Bryant 2006; Van Thieu and Koblin 2009). This attention to the effects of alcohol consumption and mechanisms of impairment reflects an increasing desire to fully address a broader scope of the epidemic in strategic ways.

A plethora of studies has demonstrated that alcohol use can impact the risk and consequences of HIV infection on a variety of levels. For example, both acute and chronic alcohol use, as well as the venues where people consume alcohol, can increase the likelihood of risky sexual behavior, thereby influencing the incidence of infection. After infection has occurred, alcohol use may hasten the progression of the disease to full-blown AIDS, for example, because alcohol-abusing patients may delay testing for infection, accessing appropriate medical care, and initiating antiretroviral therapy (ART). All of these factors can amplify the risk that the infection goes untreated and that other people are infected. Furthermore, chronic alcohol use and the presence of alcohol use disorders (i.e., alcohol abuse or dependence) also contribute to various comorbid conditions (e.g., liver disease, other co-occurring infections, or cognitive dysfunction) that have an impact on the progression of the HIV infection. Finally, alcohol interacts with many of the medications used to treat HIV infection/AIDS, and chronic alcohol use impairs patients’ adherence to ART regimens, thereby contributing to greater morbidity and mortality among the affected patients. In fact, studies found that even nonhazardous alcohol use (i.e., less than five standard drinks per drinking day) once a week or more can reduce survival of HIV-infected people by 1 year, and daily hazardous use (i.e., five or more standard drinks per day) reduces survival by 6.4 years (Braithwaite et al. 2007). To date, however, HIV/AIDS prevention and treatment strategies do not adequately take into consideration patients’ drinking behaviors. Moreover, prevention specialists and health care providers dealing with alcohol-consuming patients at risk of or already infected with HIV need additional information in order to implement effective interventions in both uninfected (i.e., HIV-negative) and infected (i.e., HIV-positive) populations.

This issue of *Alcohol Research & Health* looks at the alcohol–HIV/AIDS interaction from a variety of angles, addressing prevention issues, the physiological effects of alcohol– HIV/AIDS comorbidity, as well as treatment implications. As an introduction, this article will provide a brief overview of these topics; for more in-depth information, the reader is referred to the subsequent articles. In addition, this article highlights some suggestions for future research directions that may be able to shed more light on the underlying behavioral and biological processes in at-risk and HIV-infected individuals and will thereby help in the development of more effective prevention and treatment approaches.

Only through the development of an integrative and translational framework for HIV/AIDS and alcohol research can the devastating consequences of the HIV/AIDS epidemic be ameliorated in the future. Such a framework is presented here, which— although by no means complete— can illustrate the complexity of the interactions of alcohol use and alcohol-related behaviors with overall exposure and susceptibility to HIV infection. This framework represents an adaptation of an operations research model for HIV infection, transmission, and progression developed by Dr. Scott Braithwaite and his colleagues (pp. 280–287 in this issue). The primary aim of that model is to examine and compare the contributions of individual, group, and social–structural factors in increasing exposure and susceptibility to HIV infection and to compare the effects of interventions targeting one or more of these factors. This work extends and integrates analyses that look at simple relationships between alcohol use and sexual risk behavior to a “systems” multifactorial approach.

## Scope of the HIV/AIDS Epidemic

The population primarily affected by HIV infection and AIDS has changed since the beginning of the epidemic. According to the U.S. Surgeon General, “the epidemic has evolved from one centered on white gay men to one increasingly impacting people of color, women, and the young” (Shelton 2000, p. 1). Particularly worrisome is the increase in the number of women infected by HIV and the number who go on to develop AIDS. For example, in the United States, the proportion of women among newly reported AIDS cases increased from 7 percent in 1985 to 25 percent in 2000 (National Institute on Allergy and Infectious Diseases [NIAID] Fact Sheet HIV/AIDS 2002) and 27 percent in 2005 (CDC 2008*b*). Thus, in 2005, almost 96,000 women were living with AIDS in the United States, representing 23 percent of all AIDS cases that year (CDC 2008*b*). Particularly, non-White women now are disproportionately affected. Thus, whereas African-American and Hispanic women together represent approximately 24 percent of all U.S. women, they accounted for more than 82 percent of AIDS diagnoses for women reported in 2005 (CDC 2008*b*). Moreover, HIV/AIDS now is associated with high mortality among women, coming only behind deaths from cancer or heart disease. Thus, in 2004 it was the fifth leading cause of death among all women ages 35–44 years and the sixth leading cause of death among all women ages 25–34 years. These numbers are comparable with those found in men of the same age-groups. In Black women, the situation was even more dramatic, with HIV/AIDS being the leading cause of death among Black women ages 25–34 (compared with fourth leading cause among men of that age-group) and the third leading cause of death among Black women ages 35–44 (second leading cause in men of that age-group) in 2004 (CDC 2008*b*).

These numbers underscore the need to understand the evolving epidemic, particularly among women and minority groups, in order to develop sustainable prevention and intervention approaches. For example, the fact that more women become infected, particularly women of child-bearing age, implies that the risk of mothers transmitting the disease to their children before, during, and after birth also increases, which necessitates appropriate prevention and treatment approaches. Additional facts about the HIV/AIDS epidemic that need to be considered when developing strategies to prevent further infections and treat existing patients both in the United States and worldwide are listed in the table.

### Impact of Alcohol Use on the HIV/AIDS Epidemic

Alcohol use is common in all population subgroups in the United States, and data show that 4.4 percent of American adults meet the criteria of alcohol dependence (Grant et al. 2004). In addition, as many as 35 percent of adult drinkers may experience mild to moderate problems related to alcohol without physical dependence and therefore may be considered problem drinkers. Many of these drinkers are at risk for HIV infection, are in the early stages of infection without their knowledge and have not sought treatment but continue to drink, or have received a diagnosis of HIV/AIDS and have begun treatment with varying success. Studies also identified a strong association among women between alcohol and other drug abuse and acquisition and progression of HIV/AIDS. Thus, studies sponsored by NIAID found that in the United States, alcohol use, history of childhood sexual abuse, current domestic abuse, and use of crack/cocaine all are associated with an increased risk of heterosexual transmission of HIV (NIAID 2004). To date, however, no systematic estimates of alcohol use, abuse, and dependence using a variety of sensitive measures have been carried out in at-risk or infected populations. In an analysis of an HIV treatment population in the Veteran’s Aging Cohort Study in the United States, 35 percent of the patients were classified as currently or previously alcohol dependent (Justice et al. 2006). In Kenya, a study of people participating in a voluntary counseling and testing program (who can be considered at risk of HIV infection or may be infected) determined that more than 60 percent of the current drinkers could be classified as hazardous drinkers (Mackenzie et al. 2008). The article by Scribner and colleagues (pp. 179–183, in this issue) summarizes the existing information on the epidemiology of HIV/AIDS, including alcohol use patterns in the affected population subgroups. However, a better understanding of alcohol use patterns in at-risk and infected populations clearly is needed to more accurately define the scope of the problem and evaluate the consequences of alcohol use in these populations.

Rehm and colleagues (2009) recently related alcohol consumption to the global burden of disease and injury. The analyses demonstrated, for example, that alcohol consumption increases the risk for contracting infectious diseases, including tuberculosis (TB) and HIV/AIDS, as well as exacerbates the consequences of these diseases, such as more rapid progression, development of multidrug-resistant forms, and premature mortality from failed treatment. In absolute terms, alcohol use accounts for 13.5 percent of global mortality from infectious diseases, with the largest impact found in low-income countries with elevated rates of alcohol consumption, such as South Africa. Overall, alcohol use can contribute to HIV/AIDS deaths directly or indirectly—for example, through alcohol-related unintentional and intentional injuries, cardiovascular or hepatic damage, or neuropsychiatric disorders. These consequences of excessive alcohol use have not been assessed adequately in HIV-positive populations. Some analyses of cohorts of HIV-infected patients indicate that the role of alcohol-related hepatic and cardiovascular effects in HIV/AIDS progression and death are underappreciated and need to be addressed systematically (e.g., Conigliaro et al. 2003; also see the article by Freiberg and colleagues, pp. 237–246, in this issue). Clearly, research addressing the role of both direct and indirect alcohol effects needs to be developed further.

## Impact of Alcohol Use on HIV/AIDS Prevention

Alcohol consumption is prevalent in nearly every population group at high risk for HIV infection. In all of these groups, alcohol use can affect the risk of infection and the effectiveness of prevention efforts as well as exacerbate the consequences of an infection and progress of the disease. There are several ways to look at strategies to prevent these alcohol effects. One way is to compare behavioral prevention efforts (e.g., reduction of risky sexual behavior) and biomedical prevention efforts (e.g., vaccination or prophylactic treatment) and evaluate alcohol’s impact on both areas. A second way is to look at alcohol’s contribution at different stages of the patient’s infection process (i.e., risk of exposure to the virus, risk of infection once exposure occurs, and risk of HIV progression and organ damage once infection occurs). The following sections discuss both of these perspectives.

### Influence on Behavioral Versus Biomedical Prevention Strategies

#### Influence on Behavioral Prevention Strategies

Alcohol use may increase people’s likelihood of risky behavior and undermine the effectiveness of many prevention efforts through a variety of pathways:

- Alcohol has a direct impact on risk behaviors by impairing judgment and cognition and by disinhibiting behavior, thereby potentially increasing the likelihood of unprotected sex and other risky behaviors. In addition, alcohol-related outcome expectancies, perceived social norms, and other beliefs can influence behavior more indirectly.
- People who drink frequently also use other drugs that may enhance the risk of direct exposure to HIV, for example, by injection drug use.
- Alcohol consumption frequently occurs in settings (e.g., bars, clubs, and informal drinking places) where unprotected sex with several partners is likely to occur (Kalichman et al. 2007*b*). In general, drinking (particularly heavy drinking) is associated with risky sexual behaviors. For example, one study (Mackenzie and Kiragu 2007) reported that in a Kenyan sample, current drinkers were four times more likely to have multiple sexual partners than non-drinkers. Other studies found that alcohol-serving establishments often also are the places where sex partners meet, resulting in the formation of “sexual networks” in which HIV can spread rapidly (Chersich and Rees 2010; Weir et al. 2003). Finally, alcohol dependence may lead to trading sex for drinks, as has been reported in South Africa (see Kalichman et al. 2007*a*)
- Alcohol (like other drugs) often is a tool of sexual coercion, resulting in increased risk of HIV infection. For example, Stockman and colleagues (2009) reported that women who had been sexually coerced by use of alcohol and other drugs had a 1.5 times higher risk of having multiple sex partners than women who had not been coerced using drugs.

Based on the knowledge of these pathways, researchers have developed numerous prevention measures targeted at high-risk groups (e.g., men who have sex with men, injection drug users, or victims of sexual coercion) or high-risk settings. The article by Kalichman (pp. 184–194, in this issue) describes some of these behavioral interventions and the evidence for their effectiveness.

It is important to note, however, that despite the known effects of alcohol use on HIV transmission risk, alcohol use commonly is treated only as a background characteristic in HIV prevention measures, so that many behavioral HIV prevention interventions targeting people who drink do not seriously address alcohol use as a risk factor, thereby potentially reducing intervention effectiveness. Indeed, there are numerous examples where drinking may have reduced the potential benefits of an intervention or confounded interpretation of the results (e.g., Carey et al. 2010). Consistent with these findings, studies that have examined alcohol use as a moderator of HIV risk behavior outcomes have suggested that risk reduction interventions are more effective for participants who do not drink heavily compared with participants who report heavy drinking (e.g., Kalichman et al. 2008). Thus, future studies need to pay greater attention to the role of alcohol as a contributor to HIV transmission risk when designing and testing new prevention approaches.

#### Influence on Biomedical Prevention Strategies

Alcohol use not only may interfere with behavioral prevention approaches but also may threaten the success of emerging biomedical approaches to HIV prevention. One such approach is vaccination, and many researchers are looking for an HIV vaccine. Should such a vaccine prove effective, however, alcohol use may interfere with the person’s immunological response to the vaccination. A similar effect of alcohol abuse already has been reported in response to vaccination against the hepatitis B virus (HBV) (Hagedorn et al. 2010). Moreover, drinkers often are less connected to the health care system and therefore may be less likely to initiate vaccination.

The success of other biomedical approaches to HIV prevention, such as pre- and postexposure prophylaxis, microbicides, and others, also may be hampered by alcohol use because these approaches require proper product application and medication adherence. Recent research has demonstrated that alcohol use interferes with reliable adherence to antiretroviral medication regimens (Braithwaite et al. 2005; Glass et al. 2010; Hendershot et al. 2009), so it is likely that similar effects occur with other biomedical prevention strategies that require strict adherence, such as prophylactic treatment with HIV medications, test and treat,^1^ or microbicides. Alcohol’s interference with biomedical HIV prevention strategies is discussed in more detail in the article by Mayer and colleagues (pp. 195–202, in this issue), but it appears likely that alcohol use can reduce the effectiveness of all of these approaches. Nevertheless, current initiatives for evaluating the test-and-treat approach have not integrated behavioral interventions to address alcohol use and its relationship to nonadherence (Dieffenbach and Fauci 2009). Continuing to ignore the influence of alcohol use on response to behavioral prevention strategies as well as on adherence, however, will reduce the potential of any HIV prevention intervention.

### Influence on Prevention at Different Stages of the Infection Process

#### Reducing Risk of Exposure

**Figure 1:**
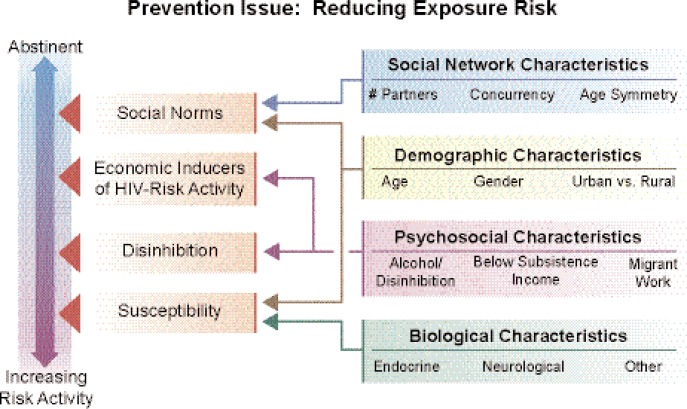
General and specific risk factors for infection. Multiple factors influenced by the use of alcohol increase the risk for HIV infection. These factors interact to increase both the general and specific contexts for infection events. Social networks, as well as demographic, psychosocial, and biological characteristics are general factors often used to target preventive interventions among alcohol users. However, these categories and “compartments” interact to influence mediators of increasing risk at the social level, such as social norms and economic inducers of HIV risk and individual-level factors related to disinhibition and biological susceptibility for infection. Scribner and colleagues (pp. 179–183, in this issue) and Kalichman (pp. 184–194, in this issue) describe the importance of these multilevel models for prevention.

Reducing people’s risk of being exposed to HIV infection obviously is important for stemming the HIV/AIDS epidemic in the United States and internationally. One approach to achieving this is using ecological models that synthesize the influences of a variety of factors underlying exposure to people with active HIV infection, particularly if they represent the interactions between these factors and alcohol use. Among the factors to be considered are the following (see figure 1 in the sidebar):

- *Social network characteristics* that include factors such as the number of partners, concurrency of alcohol and other drug use and sexual relationship, and age symmetry within a relationship, where an older HIV-positive partner may influence the risk behavior of the younger partner. All of these characteristics establish or change social norms (for a more detailed discussion, see Latkin et al. 2009).
- *Demographic characteristics* that interact with these social network characteristics and may be used to classify individuals in terms of their overall susceptibility to infection as well as their group identity (e.g., minority women, young Black men).
- *Psychosocial characteristics* that may influence drinking behaviors by altering expectations for engaging in risk behaviors, contributing to psychosocial disinhibition, and influencing settings and situations for alcohol use and risk behaviors.
- *Biological factors*, such as hormonal, neurological, and other characteristics, that also can impact risk of exposure.

Among these, social network characteristics are of particular interest because models representing social networks can help discover specific risk patterns and suggest targeted interventions. Such models may capture complex interactions between such diverse factors as alcohol levels over time in a specific geographic environment (determined using spatial mapping of specific alcohol-related venues), sex and alcohol or other drug use (using social network mapping), and individual-level characteristics. The article by Scribner and colleagues (pp. 179–183, in this issue) describes the development of such ecological models in more detail.

To interfere with this complex range of influences and thus reduce risk of exposure, interventions at multiple levels may be necessary. These may include structural interventions at the economic and policy levels, such as microeconomic interventions for women in underresourced countries that allow the women to generate sufficient income so they do not have to work as sex workers. Other interventions may be tailored to specific settings, including both formal (i.e., bars and nightclubs) and informal (i.e., “shebeens” or corner gathering places for adolescent drinking) settings in which alcohol consumption occurs and that may provide a source of employment, profit, and socializing. Of particular concern here are settings that facilitate mixing of higher-risk and lower-risk populations (e.g., cross-border settings where truck drivers rest). Interventions that have been used in these diverse settings are described in the article by Kalichman (pp. 184–194, in this issue).

#### Reducing Risk of Transmission/Infection Given Exposure

**Figure 2:**
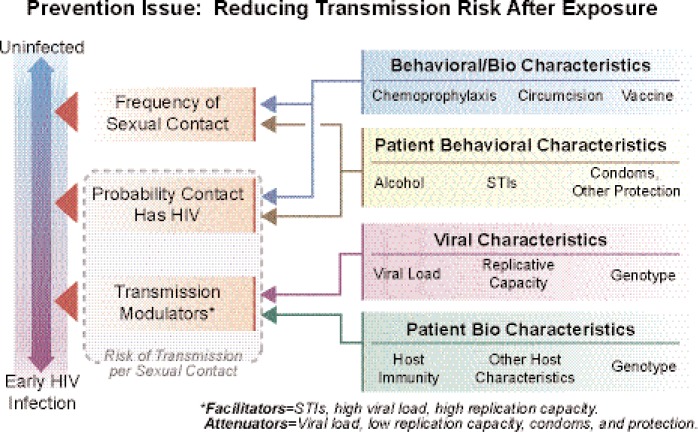
Early infection and transmission. The probability of HIV infection in any contact event is influenced by individual behavioral characteristics, such as alcohol use, the presence of other sexually transmitted infections, and condom-use behaviors. These factors influence the frequency of sexual contact with individuals of unknown HIV status and exposure to HIV. The probability of transmission may be influenced by existing and future prevention activities, such as pre-exposure prophylaxis, circumcision, or availability of vaccines. Individual behavioral and host immunological response impact transmission modulators when HIV is present. These include facilitators and attenuators in specific encounters. These factors are discussed by Pandrea and colleagues (pp. 203–218, in this issue) and future interventions by Mayer and colleagues (pp. 195–202, in this issue).

Not all people who are exposed to the virus actually become infected, and certain models are evaluating how transmission risk can be reduced. Important behavioral characteristics that augment the likelihood of infection and therefore are important targets of prevention efforts include alcohol consumption, lack of condom use or other protection (e.g., microbicides), and behaviors that result in untreated sexually transmitted infections (STIs) (see figure 2 in the sidebar). Alcohol consumption is a particularly important factor in this context because it may affect behavioral mediators of transmission (e.g., likelihood of condom or microbicide use) as well as nonbehavioral mediators (e.g., function of the person’s immune system). Therefore, research on the impact of alcohol use on the use and effectiveness of microbicides, vaccines, and preventive therapeutics has great potential to inform interventions to reduce the risk of infection. This is discussed in more detail in the article by Mayer and colleagues (pp. 195–202, in this issue). Equally important is research on the role of alcohol use in immune function impairment and in the effectiveness of agents striving to reconstitute the immune function of tissues and organs impacted by both alcohol and HIV/AIDS. In all cases, research on strategies that also can be used in settings with limited resources is of particular interest.

**Model for Alcohol and HIV/AIDS Prevention**

Complex models are being developed to describe the characteristics of the HIV epidemic and target primary, secondary, and tertiary preventive interventions. Different stages of HIV infection interact with alcohol use in different ways and represent different key prevention issues. Mixtures of populations at risk for infection, early in infection (and often unaware of their status), and those managing HIV and co-occurring diseases change the focus for prevention activities.

Current prevention models that do not include alcohol use as an environmental, social, group, and individual factor are limited in their representation of critical outcomes for the three-stage model presented here.

Another important area of study concerns approaches to reduce transmission risk that have both a behavioral and a biological component, such as prophylactic treatment with HIV medications. These medications are thought to reduce the risk of transmission if taken before or after exposure to the virus (“morning after” use). However, their use also has a behavioral component because the success of this approach depends on the patient adhering to the treatment regimen. The success of this pharmacological intervention still is under investigation; particularly, there is as yet no information on how drinkers will use this chemical prophylaxis approach. Because alcohol use is known to reduce treatment adherence and because of beliefs about the medications’ toxicity (i.e., some people report discontinuing these medications when they go to a party), it is unlikely that prophylactic treatment will be widely accepted by people with alcohol use disorders. However, because these pharmacological interventions potentially are important tools for preventing an infection in people exposed to HIV, they need to be investigated further in different patient groups.

#### Reducing Risk of Progression and Organ/Tissue Injury

**Figure 3:**
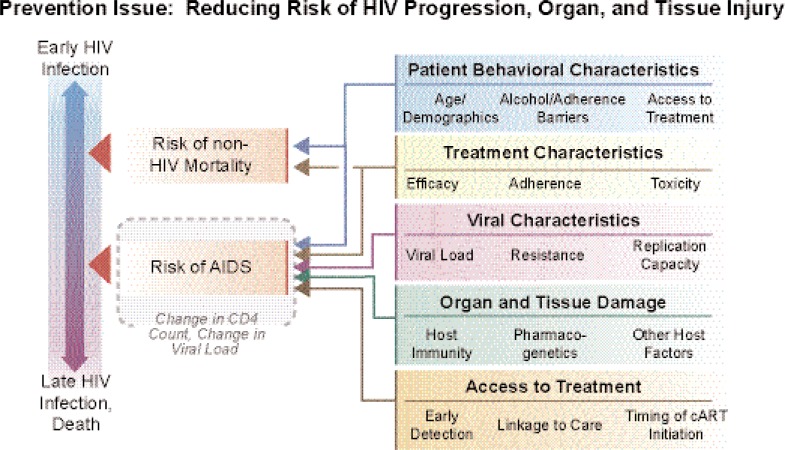
Progression and mortality. After infection, multiple factors influence organ and tissue damage and risk of mortality from both HIV and non-HIV progression and interaction. These factors include patient behavioral and biological characteristics related to treatment seeking and adherence to effective medication regimens for multiple diseases. The effective treatment for HIV/AIDS has extended patient survival, and other co-occurring chronic diseases influenced by patterns of alcohol use often are the cause of death. The special section in this volume (pp. 219–257) illustrates the potential synergistic role of alcohol for liver, lung, cardiovascular, and neurological injury and the role of adherence to complex treatment regimens in treatment management over the life course.

Once people are infected with HIV, alcohol use may impact progression of the infection and other diseases through its effects on access to and effectiveness of ART, interaction with viral characteristics, and contribution to organ and tissue damage (see figure 3 in the sidebar). Alcohol use may impede access to ART at several steps in the sequence of events that must occur for a person to begin treatment:

- Alcohol use may delay willingness to be tested for HIV, increasing the likelihood that infection will only be detected after serious HIV-related disease develops.
- Alcohol use may make it less likely that people with a diagnosed HIV infection link with appropriate care and show up for regular appointments; these people therefore are less likely to be considered suitable candidates for starting ART.
- Alcohol-abusing people may be less willing to start ART because of concerns over symptoms, toxicity, or anticipated adherence problems.

All of these factors limit access to ART and therefore contribute to the risk of HIV/AIDS progression and death.

In addition to these effects on ART and its effectiveness, alcohol abuse also has been shown to accelerate and magnify the organ and tissue damage resulting from the HIV infection and from comorbid diseases. Some of the acute and chronic interactions of alcohol with HIV/AIDS progression and associated conditions are reviewed in more detail in the article by Pandrea and colleagues (pp. 203– 218, in this issue). Because the life expectancy of HIV-infected people has been prolonged as a result of ART, comorbid conditions, such as hepatic, pulmonary, neurological, cardiovascular, and metabolic diseases; certain tumors; and other clinical manifestations associated either with long-term HIV or prolonged ART, have assumed greater importance as causes of morbidity and mortality in HIV-positive people. Most of these conditions, but particularly liver disease and neurological disorders, can be exacerbated by alcohol abuse and dependence. For example, ART frequently has hepatotoxic effects; moreover, many HIV-positive patients also are infected with hepatitis B and C viruses that also damage the liver (Goedert et al. 2002). Chronic alcohol consumption also causes liver injury and may therefore accelerate the development of serious liver disease in patients coinfected with HIV and hepatitis viruses. Thus, understanding liver disease progression in the context of HIV infection and alcohol abuse is becoming an important issue in caring for these patients. For example, research is needed on the response of alcohol-abusing HIV patients to treatment of liver disease. These and other issues are reviewed in the article by Barve and colleagues (pp. 229–236, in this issue).

Another area of growing concern among HIV-positive patients is neurological injury. Both HIV infection and alcohol abuse have detrimental effects on brain function, which are reviewed in the article by Rosenbloom and colleagues (pp. 247–257, in this issue). Another common neurological disorder is peripheral neuropathy. This typically painful and debilitating condition, which is characterized by inflammation or degeneration of nerves outside the brain and spinal cord, severely compromises quality of life and productivity. It is found in approximately 30 percent of HIV-infected patients and almost 100 percent of AIDS cases (Ferrari et al. 2006). Similarly, peripheral neuropathy is the most common neurological complication in alcoholism (Diamond and Messing 1994). Although both HIV infection and alcohol abuse can induce peripheral neuropathy, they may do so through different mechanisms, and this issue requires further investigation.

Research also is needed to characterize the synergistic interactions of alcohol, HIV infection, and ART in other body systems. For example, as described by Drs. Freiberg and Kraemer (pp. 237–246, in this issue), these interactions can play a significant role in the development of cardiovascular disease. Similarly, lung health also can be impaired in alcohol-abusing HIV-infected patients, as explained in the article by Quintero and Guidot (pp. 219–28, in this issue). Research that will lead to a better understanding of the effects of alcohol on various HIV-associated comorbidities is a high priority and may be promoted by the emerging field of systems biology. In addition, translational and clinical studies are needed to translate basic research results into improved strategies for preventing and treating these HIV-associated comorbidities and their consequences.

## Treatment of Concurrent HIV/AIDS and Alcohol Problems

Treatment of patients with concurrent HIV/AIDS and alcohol problems is complicated and often ineffective. As mentioned earlier and further described in the article by Drs. Braithwaite and Bryant (pp. 280–287, in this issue), alcohol-abusing patients exhibit reduced adherence to their treatment regimens, which typically have to be followed exactly in order to be most effective. Failure to adhere to the regimen enhances the likelihood that the virus becomes resistant to the medications used and that HIV disease progresses more rapidly. At the same time, alcohol can interact with the HIV medications and may exacerbate the toxic effects associated with the long-term use of some of these agents. Fear of this toxicity leads many patients to stop taking their medications when they know they will be drinking or discourage them from initiating treatment at all.

A second aspect of the comorbidity of HIV/AIDS and alcohol abuse or dependence is whether alcoholism treatment should be initiated as part of the HIV/AIDS intervention. Alcoholism therapy requires substantial investments of time, effort, and expense also at the part of the patient, which may seem unreasonable to patients with less severe drinking problems. In addition, it is conceivable that patients may be afraid of stigmatization if they seek treatment for problem drinking, whereas seeking treatment for a medical condition may be considered more acceptable. Finally, many problem drinkers at early stages of change may not think they have a drinking problem and therefore do not think they require treatment. Thus, perceptions regarding the need for and usefulness of treatment for problem drinking may keep many affected patients from seeking treatment in the first place. Even if treatment is initiated, which typically involves behavioral approaches, the effectiveness of these interventions often is limited, as described in the article by Samet and Walley (pp. 267–279, in this issue).

These considerations underscore the need to develop pharmacological and low-threshold interventions, particularly for patients who are not prepared to invest in formal alcohol treatment or who need to seek treatment outside of the usual alcohol treatment settings (e.g., in HIV/AIDS testing and treatment settings). Moreover, new approaches are needed for effectively encouraging problem drinkers to initiate treatment—for example, by modifying their perceptions of the acceptability of their drinking as well as of the attractiveness of various intervention options for people at risk of HIV infection, of unknown HIV status, or with active HIV infection. Finally, medications for alcohol-abusing and alcohol-dependent HIV-positive patients are needed that ideally impact drinking behavior as well as improve the patient’s immune function and decrease progression of the HIV infection. Chronic alcohol use is associated with impairment of the immune system. Among other effects, alcohol impairs the functioning of the exact cells that are the targets of HIV—cells called CD4 T-lymphocytes and macrophages. Thus, it would be desirable to have medications that inhibit the alcohol-mediated spread of the HIV infection among the CD4 T-lymphocytes and which at the same time address the underlying alcohol disorder, thereby enhancing adherence to ART. In fact, such an effect has been observed with the medication naltrexone, which is used in the treatment of alcoholism (Wang et al. 2006). However, additional treatment options are needed, and novel pharmacologic approaches to the treatment of disorders affecting the immune system have been suggested (Tuluc et al. 2009).

## Future Research

Alcohol use is one of the most modifiable factors contributing to the risk of HIV infection as well as to the progression of HIV/AIDS. Past research has shown the importance of alcohol use and misuse in the acquisition, progression, and transmission of HIV disease. Even low levels of alcohol use in HIV-positive people may lead to accelerated disease progression. Moreover, alcohol use that occurs in high-risk situations and modifies risk-taking behavior is likely to lead to increased rates of transmission. Accordingly, elucidation of the interaction between the behavioral actions and biological processes by which alcohol contributes to increased disease susceptibility and progression is necessary for developing tailored interventions to be used with individuals, groups, and environments where the risk for infection and transmission is high. These interventions need to act at multiple levels to ensure their effectiveness.

The complex and global nature of unresolved questions surrounding the relationship between alcohol and HIV/AIDS indicates the need for a multidisciplinary approach to research. Thus, investigators representing a broad array of academic disciplines and engaged in cross-cutting fields of science need to work together to design hypotheses-driven studies that utilize rigorous methodologies from epidemiological, basic, clinical, and behavioral research. In particular, biomedical research that can lead to improved treatments for people with co-occurring HIV/AIDS and alcohol use disorders needs to be strengthened. This also can help prevent the spread of HIV/AIDS, particularly from mothers to their children (i.e., vertical transmission), which is an increasing threat because of the growing numbers of women with HIV disease.

### Focus on Integrating Behavioral and Biological Research

One important focus of any alcohol– HIV research needs to be the integration of basic biological and behavioral research in order to address both the behavioral aspects contributing to the spread of HIV infection (e.g., risky sexual behavior or reduced adherence to ART in drinkers) and the biological factors exacerbating infection risk and disease progression (e.g., alcohol-related impairment of the immune system or damage to other organs and tissues). These findings then need to be translated into effective prevention and treatment strategies for people whose drinking places them at risk of adverse HIV-related outcomes, such as increased susceptibility to infection, more rapid disease progression, treatment complications, and increased mortality. In particular, this research needs to assess and strive to improve the overall impact of prevention and treatment interventions focusing on alcohol misuse in the target populations because, as shown in the article by Samet and Walley (pp. 267–279 in this issue), the effectiveness of such interventions so far still is limited. Some investigators have proposed conceptual models that integrate multiple concurrent epidemics (i.e., syndemic models) for the prevention and treatment for HIV; however, these models still need to be developed further to reflect the different states of knowledge of multiple factors at many levels of analysis that currently still exist in the field. Nevertheless, such operations research models can help to more systematically identify the most promising starting points for developing and implementing effective interventions in specific at-risk populations, thereby helping to optimize limited research and intervention resources.

This research also needs to take into consideration the different needs of patients in different countries. For example, in the United States one focus of the research is on long-term treatment of HIV to prevent disease progression and reduce the risk of transmission to other people by lowering the viral load of patients. In underresourced countries, in contrast, treatment availability is limited, and the emphasis needs to be placed on strategies to prevent drinking and/or risky sexual behavior in the populations at highest risk of HIV infection. Particularly in settings where resources for prevention and treatment are highly limited, neglecting to address alcohol use (the most common form of substance abuse in those settings) and its association with acquisition of HIV as well as treatment failure can be a costly error in the longer term.

### Focus on Translational Research

The lack to date of attention to alcohol use as a mediating factor results at least in part from deficiencies in translational research—the gap between research on these interrelationships and development of appropriate interventions on the one hand and the actual implementation of prevention and treatment strategies in real-world settings on the other hand. This gap is only now beginning to be addressed (Hirschhorn et al. 2007). Translational research stresses the need for developing a research context for implementing and disseminating effective interventions in combination with one another. For example, models are needed to systematically identify choice points for developing, testing, and disseminating effective intervention strategies, in the process incorporating a wide range of factors related to alcohol use. Such an effective translational research agenda also requires a pool of qualified investigators, an academic culture that fosters collaboration between clinical and basic science researchers, institutional structures that support interdisciplinary programs, and appropriate administrative and regulatory processes.

## Conclusion

Despite 30 years of research, HIV/AIDS still is one of the greatest health challenges worldwide and continues to take an enormous toll both in terms of human suffering and mortality and with respect to the associated economic costs. Alcohol use and misuse continues to play an important role in the acquisition, progression, and transmission of HIV. Therefore, in order to reduce the number of new HIV cases and improve the prognosis of those already infected with the virus, it is essential to bring the two fields of alcohol research and HIV research together to allow for advances in both prevention and treatment.

The articles in this issue of *Alcohol Research & Health* present specific findings from current research into the interactions of alcohol use and HIV/AIDS, providing an overview of behavioral and biomedical prevention approaches, the physiological consequences of concurrent alcohol use and HIV infection on the body, and treatment challenges and strategies to address both conditions. Some articles summarize research and offer models from relatively broad problem areas, whereas others focus on specific problems that to date have received little attention. Together, the articles provide a foundation for a discussion of future research and new directions in these areas. Although this journal issue can only represent a few of the multiple approaches to the problems associated with alcohol use and HIV/AIDS, it begins to sketch out the current way of thinking about the relationship of alcohol use to the HIV epidemic both in the United States and abroad.

Much has been learned about this relationship in the past three decades. For example, it is now clear that it takes a combination of medications to control the infection; similarly, it may take a such a “cocktail approach” combining vaccines and behavioral interventions to prevent the acquisition and transmission of HIV infections. The challenge for researchers and clinicians now is to understand how to develop the optimal combination of preventive and therapeutic measures that remain effective over time. Meeting this challenge will require the development of broad models that incorporate all relevant perspectives; this can only be achieved by intensive and multidisciplinary collaboration between the alcohol and HIV/AIDS fields. ?

## Figures and Tables

**Table:** Facts About the HIV/AIDS Epidemic in the United States and Worldwide

**United States**
Roughly 1 million people were living with HIV/AIDS in the United States at the end of 2003 (CDC 2008*c*). Since the start of the AIDS epidemic, 1.5 million Americans have been infected with HIV and more than 524,000 have died of AIDS. In 2006, the number of new HIV infections in the United States was approximately 56,300 (CDC 2008*a*). In 2006, African Americans account for 49 percent of new HIV infections (CDC 2008*c*). In 2004, HIV/AIDS was the leading cause of death for African-American women ages 25–34 (CDC 2008*b*) The number of women living with HIV has tripled in the past two decades. In 2006, more than half of all new infections were among people ages 25–44 (CDC 2008*c*). By 2015, more than half of people living with HIV/AIDS will be over 50 years old.
**Global Epidemic**
At the end of 2008, more than 33 million people were living with HIV (UNAIDS 2009). In 2008, approximately 2.7 million people were newly infected with HIV, corresponding to about 7,400 people every day (UNAIDS 2009). In 2008, approximately 2 million people died from AIDS, corresponding to about 5,500 people every day (UNAIDS 2009). In 2008, one child died from AIDS every 2 minutes (UNAIDS 2009). Sub-Saharan Africa remains the most heavily affected region, accounting for 68 percent of HIV infections worldwide and 72 percent of the world’s AIDS-related deaths in 2008 (UNAIDS 2009). Worldwide, more than 15 million children have lost one or both parents to AIDS; this number is expected to increase to 20 million by 2010 (Global AIDS Alliance 2009).

## Footnotes

1 The term test and treat refers to a strategy whereby people are aggressively tested for HIV infection and are started on antiretroviral medications as soon as they test positive for the virus, with the goal of reducing the amount of virus in the blood and thereby preventing transmission of the infection to others. To date, many people do not know they are infected because they are not regularly tested, and antiretroviral medications typically are started only once the patient begins to exhibit more severe symptoms of the infection.

## References

[b1-arh-33-3-167] Braithwaite RS, Conigliaro J, Roberts MS (2007). Estimating the impact of alcohol consumption on survival for HIV^+^ individuals. AIDS Care.

[b2-arh-33-3-167] Braithwaite RS, McGinnis KA, Conigliaro J (2005). A temporal and dose-response association between alcohol consumption and medication adherence among veterans in care. Alcoholism: Clinical and Experimental Research.

[b3-arh-33-3-167] Bryant KJ (2006). Expanding research on the role of alcohol consumption and related risks in the prevention and treatment of HIV/AIDS. Substance Use and Misuse.

[b4-arh-33-3-167] Carey MP, Senn TE, Vanable PA (2010). Brief and intensive behavioral interventions to promote sexual risk reduction among STD clinic patients: Results from a randomized controlled trial. AIDS and Behavior.

[b5-arh-33-3-167] Centers for Disease Control and Prevention (CDC) HIV incidence [article online], 2008a. http://www.cdc.gov/hiv/topics/surveillance/incidence.htm.

[b6-arh-33-3-167] Centers for Disease Control and Prevention (CDC) (2008b). HIV/AIDS among women. CDC HIV/AIDS Fact Sheet.

[b7-arh-33-3-167] CDC (2008c). HIV and AIDS in the United States: A picture of today’s epidemic. CDC Fact Sheets [article online].

[b8-arh-33-3-167] Chersich MF, Rees HV (2010). Causal links between binge drinking patterns, unsafe sex and HIV in South Africa: It’s time to intervene. International Journal of STD & AIDS.

[b9-arh-33-3-167] Conigliaro J, Gordon AJ, McGinnis KA (2003). How harmful is hazardous alcohol use and abuse in HIV infection: Do health care providers know who is at risk?. Journal of Acquired Immune Deficiency Syndromes.

[b10-arh-33-3-167] Diamond I, Messing RO (1994). Neurological effects of alcoholism. Western Journal of Medicine.

[b11-arh-33-3-167] Dieffenbach CW, Fauci AS (2009). Universal voluntary testing and treatment for prevention of HIV transmission. JAMA: Journal of the American Medical Association.

[b12-arh-33-3-167] Doran D (2009). AIDS deaths top 25 mln but infections slow [article online]. http://www.google.com/hostednews/afp/article/ALeqM5hxc-xJF5Wv4ROHi6TPUcqiuqVrmA.

[b13-arh-33-3-167] Ferrari S, Vento S, Monaco S (2006). Human immunodeficiency virus-associated peripheral neuropathies. Mayo Clinic Proceedings Mayo Clinic.

[b14-arh-33-3-167] Glass TR, Battegay M, Cavassini M (2010). Longitudinal analysis of patterns and predictors of changes in self-reported adherence to antiretroviral therapy: Swiss HIV Cohort Study. Journal of Acquired Immune Deficiency Syndromes.

[b15-arh-33-3-167] Global AIDS Alliance (2009). Protect the children [article online].

[b16-arh-33-3-167] Goedert JJ, Eyster ME, Lederman MM (2002). End-stage liver disease in persons with hemophilia and transfusion-associated infections. Blood.

[b17-arh-33-3-167] Grant BF, Dawson DA, Stinson FS (2004). The 12-month prevalence and trends in DSM-IV alcohol abuse and dependence: United States, 1991–1992 and 2002–2002. Drug and Alcohol Dependence.

[b18-arh-33-3-167] Hagedorn HJ, Rettmann NA, Dieperink EW (2010). Antibody response to hepatitis B vaccine in substance use disorder patients. Drug and Alcohol Dependence.

[b19-arh-33-3-167] Hendershot CS, Stoner SA, Pantalone DW, Simoni JM (2009). Alcohol use and antiretroviral adherence: Review and meta-analysis. Journal of Acquired Immune Deficiency Syndrome.

[b20-arh-33-3-167] Hirschhorn LR, Ojikutu B, Rodriquez W (2007). Research for change: Using implementation research to strengthen HIV care and treatment scale-up in resource-limited settings. Journal of Infectious Diseases.

[b21-arh-33-3-167] Justice AC, Lasky E, McGinnis KA (2006). Medical disease and alcohol use among veterans with human immunodeficiency infection: A comparison of disease measurement strategies. Medical Care.

[b22-arh-33-3-167] Kalichman SC, Simbayi LC, Kaufman M (2007a). Alcohol use and sexual risks for HIV/AIDS in sub-Saharan Africa: Systematic review of empirical findings. Prevention Science.

[b23-arh-33-3-167] Kalichman SC, Simbayi LC, Cain D, Jooste S (2007b). Alcohol expectancies and risky drinking among men and women at high-risk for HIV infection in Cape Town South Africa. Addictive Behaviors.

[b24-arh-33-3-167] Kalichman SC, Simbayi LC, Vermaak R (2008). Randomized trial of a community-based alcohol-related HIV risk-reduction intervention for men and women in Cape Town, South Africa. Annals of Behavioral Medicine.

[b25-arh-33-3-167] Latkin C, Donnell D, Celentano DD (2009). Relationship between social norms, social network characteristics, and HIV risk behaviors in Thailand and the United States. Health Psychology.

[b26-arh-33-3-167] Mackenzie C, Kiragu K, Odingo G (2008). Is it feasible to integrate alcohol-related risk reduction counseling into VCT Services? Findings from Kenya. Horizons Final Report.

[b27-arh-33-3-167] Mackenzie C, Kiragu K Integrating Alcohol Risk Reduction Counseling Into VCT Services in Kenya: Preliminary Evaluation Results.

[b28-arh-33-3-167] National Institute of Allergy and Infectious Diseases (NIAID) (2002). HIV/AIDS Statistics.

[b29-arh-33-3-167] NIAID (2004). Women’s Health in the United States: Research on Health Issues Affecting Women.

[b30-arh-33-3-167] Rehm J, Mathers C, Popova S (2009). Global burden of disease and injury and economic cost attributable to alcohol use and alcohol-use disorders. Lancet.

[b31-arh-33-3-167] Shelton DA (2000). Changing demographics in HIV. American Medical News.

[b32-arh-33-3-167] Stockman JK, Campbell JC, Celentano DD (2010). Sexual violence and HIV risk behaviors among a nationally representative sample of heterosexual American women: The importance of sexual coercion. Journal of the Acquired Immune Deficiency Syndromes.

[b33-arh-33-3-167] Tuluc F, Lai JP, Kilpatrick LE (2009). Neurokinin 1 receptor isoforms and the control of innate immunity. Trends in Immunology.

[b34-arh-33-3-167] UNAIDS (2009). AIDS Epidemic Update: November 2009.

[b35-arh-33-3-167] Van Tieu H, Koblin BA (2009). HIV, alcohol, and noninjection drug use. Current Opinion in HIV and AIDS.

[b36-arh-33-3-167] Wang X, Douglas SD, Peng JS (2006). Naltrexone inhibits alcohol-mediated enhancement of HIV infection of T lymphocytes. Journal of Leukocyte Biology.

[b37-arh-33-3-167] Weir SS, Pailman C, Mahlalela X (2003). From people to places: Focusing AIDS prevention efforts where it matters most. AIDS.

